# A virtual experimenter to increase standardization for the investigation of placebo effects

**DOI:** 10.1186/s12874-016-0185-4

**Published:** 2016-07-18

**Authors:** Bjoern Horing, Nathan D. Newsome, Paul Enck, Sabarish V. Babu, Eric R. Muth

**Affiliations:** Department of Psychology, Clemson University, Clemson, SC USA; Human-Centered Computing Division, School of Computing, Clemson University, Clemson, SC USA; Department of Psychosomatic Medicine and Psychotherapy, University Hospital Tübingen, Tübingen, Germany

**Keywords:** Virtual reality, Placebo mechanisms, Placebo analgesia, Bias, Blinding, Standardization

## Abstract

**Background:**

Placebo effects are mediated by expectancy, which is highly influenced by psychosocial factors of a treatment context. These factors are difficult to standardize. Furthermore, dedicated placebo research often necessitates single-blind deceptive designs where biases are easily introduced. We propose a study protocol employing a virtual experimenter – a computer program designed to deliver treatment and instructions – for the purpose of standardization and reduction of biases when investigating placebo effects.

**Methods:**

To evaluate the virtual experimenter’s efficacy in inducing placebo effects via expectancy manipulation, we suggest a partially blinded, deceptive design with a baseline/retest pain protocol (hand immersions in hot water bath). Between immersions, participants will receive an (actually inert) medication. Instructions pertaining to the medication will be delivered by one of three metaphors: The virtual experimenter, a human experimenter, and an audio/text presentation (predictor “Metaphor”). The second predictor includes falsely informing participants that the medication is an effective pain killer, or correctly informing them that it is, in fact, inert (predictor “Instruction”). Analysis will be performed with hierarchical linear modelling, with a sample size of *N* = 50. Results from two pilot studies are presented that indicate the viability of the pain protocol (*N* = 33), and of the virtual experimenter software and placebo manipulation (*N* = 48).

**Discussion:**

It will be challenging to establish full comparability between all metaphors used for instruction delivery, and to account for participant differences in acceptance of their virtual interaction partner. Once established, the presence of placebo effects would suggest that the virtual experimenter exhibits sufficient cues to be perceived as a social agent. He could consequently provide a convenient platform to investigate effects of experimenter behavior, or other experimenter characteristics, e.g., sex, age, race/ethnicity or professional status. More general applications are possible, for example in psychological research such as bias research, or virtual reality research. Potential applications also exist for standardizing clinical research by documenting and communicating instructions used in clinical trials.

## Background

To a large extent, placebo effects arise from the psychosocial setting in which a treatment is delivered, most notably the physician/patient or experimenter/participant interaction [1]. These effects can be substantial [2, 3]. However, it is difficult to systematically tease apart the contributions made by separate aspects of an interaction. In this paper, we propose the use of a virtual experimenter as a platform to address these issues with minimized risk of bias.

We define the placebo effect (PE) as that part of an actually experienced symptom improvement mediated by psychological expectancy concerning a treatment, regardless of the treatment’s actual efficacy [4]. This expectancy arises from multiple sources in the treatment’s psychosocial context, such as verbal instruction [5], conditioning [6] or social learning [7].

PEs are most well known in the context of randomized clinical trials, where the placebo group receives an inert treatment to serve as control for the actual treatment [8]. However, the change happening in the placebo group is always a composite measure, including the actual placebo effect and numerous other factors [9, 10]. Some of these are a feature of the nosological subject matter, such as the natural course of disease and the fact that people tend to seek out treatment at peaks of symptom severity. These factors can lead to regression to the mean, which describes the fact that extreme values will tend to converge toward less extreme values at retest. Other factors include distinct psychological phenomena, like response biases, which cause study participants to modify their answering behavior after receiving a placebo treatment, without actually affecting their symptom experience [9, 10].

To investigate PEs per se, dedicated research involving additional control groups has been performed. Different from classical clinical trials where the placebo group is the control group, dedicated placebo research inquires into the conditions in which PEs occur, and the mechanisms involved [11]. A number of paradigms exist which employ active treatments [11, 12]. For example, in the “balanced placebo design” [13], the actual treatment (active vs. inert) is paired with the information with which it is administered (“active” vs. “inert”). Like double-blind clinical trials, this permits blinding. However, use of active treatment is often precluded due to ethical and regulatory obstacles. Especially when investigating healthy participants to identify basal mechanisms of the placebo response, this frequently leads to the employment of more basic designs. One of these designs is a single-blind deceptive placebo design. For example, in one of our own studies [14], two groups receiving the same inert treatment were compared, with one of them being (deceptively) told that the treatment is actually effective, and one being (truthfully) told that it is not. Under these circumstances, the above mentioned psychological confounds can be exacerbated. Not only are the investigated variables highly affected by psychological influences to begin with, but additionally, blinding of the experimenter is frequently impossible or impractical [10]. Consequently, while great progress has been made in identifying the neuropharmacological [15], neuronal [16] and even genetic [17] underpinnings of placebo responsiveness, some of this research stands on shaky foundations [10]. If the experimental interaction is not tightly controlled, it is difficult if not impossible to establish causation in placebo trials: The reported symptom change of a participant cannot merely be attributed to the (intentional) treatment difference, but possibly to subtle but systematic (unintentional) differences in the broader experimental context. These differences contribute to a mean difference of experimental group versus control group. Some of these context factors have been explored, such as experimenter variables like a reassuring, positive attitude [18], empathy [1], affect-oriented communication [19], professional status [20] or sex [21]. However, these studies have been rarely replicated.

**Fig. 1:**
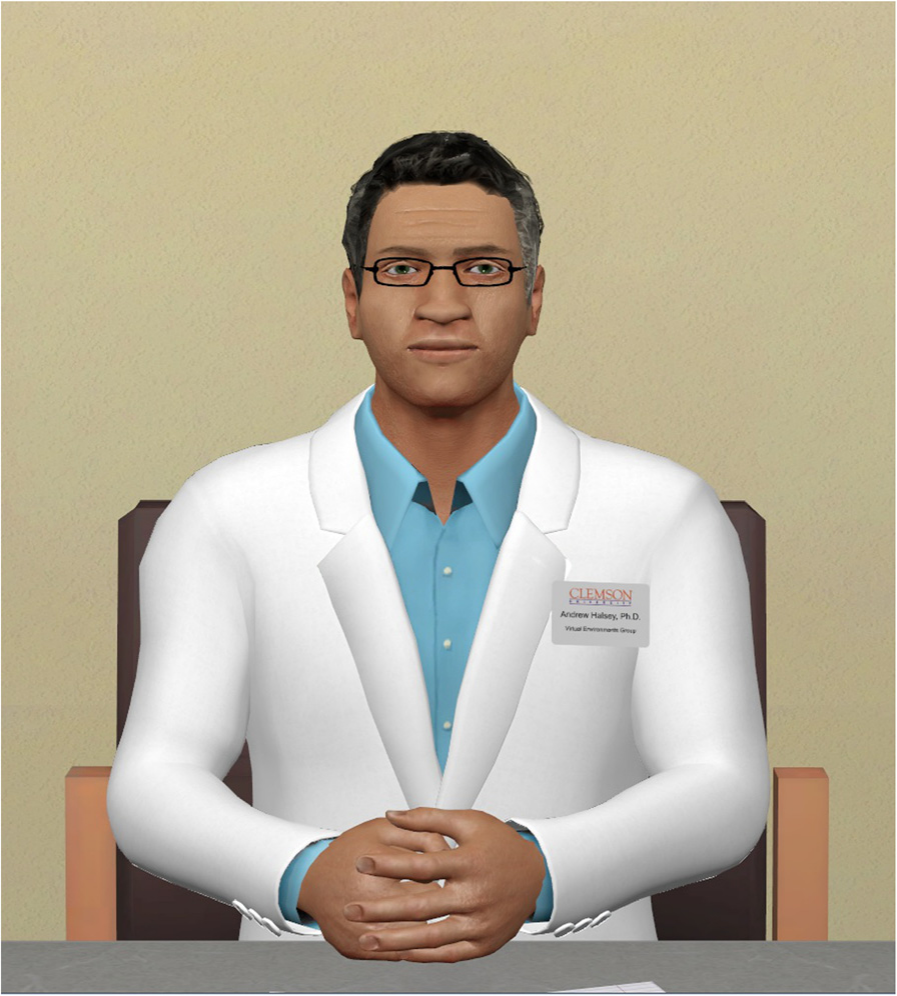
“Dr. Halsey”, the virtual experimenter

We propose that some of the limitations of research addressing psychological entities like symptom experiences could be overcome by the use of a virtual experimenter (VEx; Fig. 1). The VEx is a computer program designed to perform the function of randomized treatment allocation and treatment delivery. While preserving or even exceeding psychosocial characteristics of a conversation [22] (which are required for PEs to arise in the first place), it would perform social tasks with complete standardization – this is in contrast to pre-recorded messages, which do not induce the same extent of social presence as an embodied conversational agent.

From this framework, it is easy to manipulate aspects of the instruction or the appearance of the VEx without changing any of the other parameters, as would almost necessarily be the case if the task were performed by a human experimenter (HEx). While virtual reality has been used in some related applications before (e.g., as “virtual reality-based analgesic” distraction techniques [23] or interview partners [24] in the scope of psychological research), its use for the proposed purpose has not been investigated.

In the following, we present a comprehensive study protocol to test the VEx’ efficacy in eliciting PEs, to establish its viability as a substitute for treatment allocation and delivery by a HEx (section “Methods”). Due to the well-documented efficacy of placebo on pain (placebo analgesia), a pain modality will be employed as the method for symptom induction. The protocol combines several features (virtual reality software and pain protocol). These components have been separately investigated in pilot studies presented following the proposed protocol (section “Current status”). We outline the creation of the VEx under “Development of the stimulus material”, and its evaluation under “Pilot study 1”. To assess reproducibility and possible carry-over effects, we conducted a pilot study of the pain protocol, as well. Core findings are presented under “Pilot study 2”.

## Methods

We propose the following methods to determine the VEx’ efficacy in inducing PEs. The interaction with the VEx will be compared to an HEx interaction, and an audio/text based interaction. All non-interventional interactions such as the pain testing will be performed by a blinded laboratory assistant.

### Design

The study will follow a partially blinded, deceptive, repeated measures design. Participants will undergo a baseline pain measurement (five 60 s immersions of the hand in hot water; see “Pain protocol”), followed by treatment with a supposed pain killer, and a retest pain measurement. In this design, every participant will only receive an oral placebo (no active medication), but groups will differ as to the instruction about the (actually inert) “medication’s” efficacy.

Within-persons and between-persons predictors include

- ■ the mode of treatment/instruction presentation (between-persons predictor Metaphor, 3 levels: HEx, VEx, text/audio)
- ■ the instruction itself (between-persons predictor Instruction, 2 levels: “medication” (deceptive) vs. “inert” (truthful)); this manipulation is double-blind to both participant and laboratory assistant
- ■ the pain measurement repetition, which constitutes nested within-persons predictors (immersion Time nested in immersion Trials nested in immersion Sequences). In the scope of this study outline, we will only address Trials and Sequences pertaining to the expected results.

**Fig. 2:**
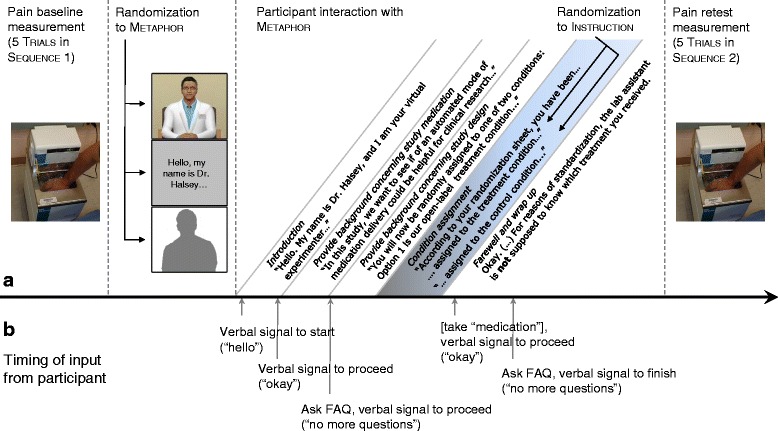
Experimental design (A) and participant verbal input during the interaction (B). **a** Between pain baseline and retest, participants will be randomly assigned to Metaphor and Instruction. Different sections of the participant interaction with Metaphor (*Introduction* through *Farewell and wrap up*) are displayed. The shaded area (*Condition assignment*) corresponds to allocation to Instruction. **b** Corresponding to the participant interaction sections, the arrows indicate the time points of verbal input required for the instructions to proceed

See Fig. 2a for a design and protocol outline. Within-persons and between-persons predictors correspond to level 1 and level 2 variables used in hierarchical linear modelling nomenclature (see “Analysis and sample size estimation”).

### Study population

Participants will be recruited from Clemson University's student population, staff and faculty. Exclusion criteria will be applied (Table 1). We will balance the sample for sex/gender and will attempt mean matching for age, to yield roughly equal distributions in all combinations of the two between-persons predictors. Both sex/gender and age are possible confounds, as they may influence pain sensitivity [25, 26], placebo responsiveness [27] or the reception of the technology involved in the intervention [28].

**Table 1:** Inclusion criteria for the proposed study

18 < Age < 64	
No past or present brain or nerve conditions (e.g. fainting, stroke, neuropathy)	
No past or present pain disorder (e.g. diagnosed migraine, chronic back pain)	
No open wounds or skin conditions on the dominant hand (e.g. hangnail, graze)	
Abstained from recreational drugs (alcohol, cannabis) for at least 24 h before the lab visit	
Abstained from pain medication (and other medication feasibly affecting experimental parameters) for at least 24 h before the lab visit	

Due to the deceptive nature of the protocol, full informed consent cannot be procured prior to participation; instead, a cover story will be presented to the participants. After the experiment, participants will receive full disclosure of the manipulation. Express permission will be asked of them to consider their data in the analysis. They will be given the choice to withdraw their data from analysis at any point prior to publication, without the need for justification.

### Procedure

Participants will continually rate their pain during Sequence 1 under supervision of a laboratory assistant. They will then be randomly assigned to one of the 3(Metaphor)*x*2(Instruction) experimental groups. In a separate room, they will interact with the Metaphors delivering the Instructions. For the VEx and audio/text condition, interactional components are displayed in Fig. 2b. Crucially, neither assignment will be revealed to the laboratory assistant.

During the placebo manipulation, the inert medication will be automatically dispensed using a self-constructed pill dispenser controlled by an Arduino platform (https://www.arduino.cc/). For VEx and audio/text condition, pill dispension will be performed by the presentation software; in the HEx condition by pressing a button.

An overall inter-Sequence interval of 35 min will be observed for cutaneous sensitivity to recover, and to provide a plausible cover story for the medication to take effect (in the “medication” instruction).

The pain change at retest, compared between experimental groups, will be the main outcome.

### Pain protocol

For symptom induction, we will employ a hot water bath (RTE-111, Neslab Instruments, Inc., Newington, NH), which is an established method for painful stimulation [29, 30]. Tonic heat pain has been successfully employed in the context of placebo studies with comparable stimulus strengths [31, 32]. Participants will be asked to immerse their dominant hand in the water kept at a constant temperature of 47 °C, which is painful for most people [33]. They will be informed that they can discontinue the experiment at any time. Two familiarization trials will be given where participants briefly put their hand in and out of the water. They will then be asked to immerse their hand for 1 min, take it out for 30 s, put it back in for 1 min etc., for a maximal number of 5 immersions (total immersion time 5 min). Immersion and removal will be prompted by automated verbal displays on screen, and accompanying audio signals.

Throughout the sequence, participants will continually rate their pain experience with a 0 to 100 digital visual analogue scale (VAS) displayed on a touch screen. They will be asked to leave their finger on the screen and move it once their pain perception changes.

After an interval of 35 min, another sequence of 5 immersions will be performed. After each sequence, they will also give a single retrospective rating of pain intensity and unpleasantness (VAS from 0 to 100) to establish comparability to conventional non-continuous measures.

### Predictor Metaphor: virtual experimenter, human experimenter and audio/text condition

Identical instructions will be delivered by the three Metaphors. They will consist of a brief introduction of the agent delivering the instructions (VEx, HEx or disembodied voice), a cover story explaining the (actually fictional) background of the experiment, and the treatment allocation constituting the placebo manipulation. Participants will also interact with the VEx and audio/text condition in the scope of a frequently-asked questions (FAQ) module; the HEx will also provide the list of FAQs and will attempt to restrict the interaction to these. The questions revolve mostly around aspects of the experimental protocol, like they would in a naturalistic conversation with an HEx.

**Fig. 3:**
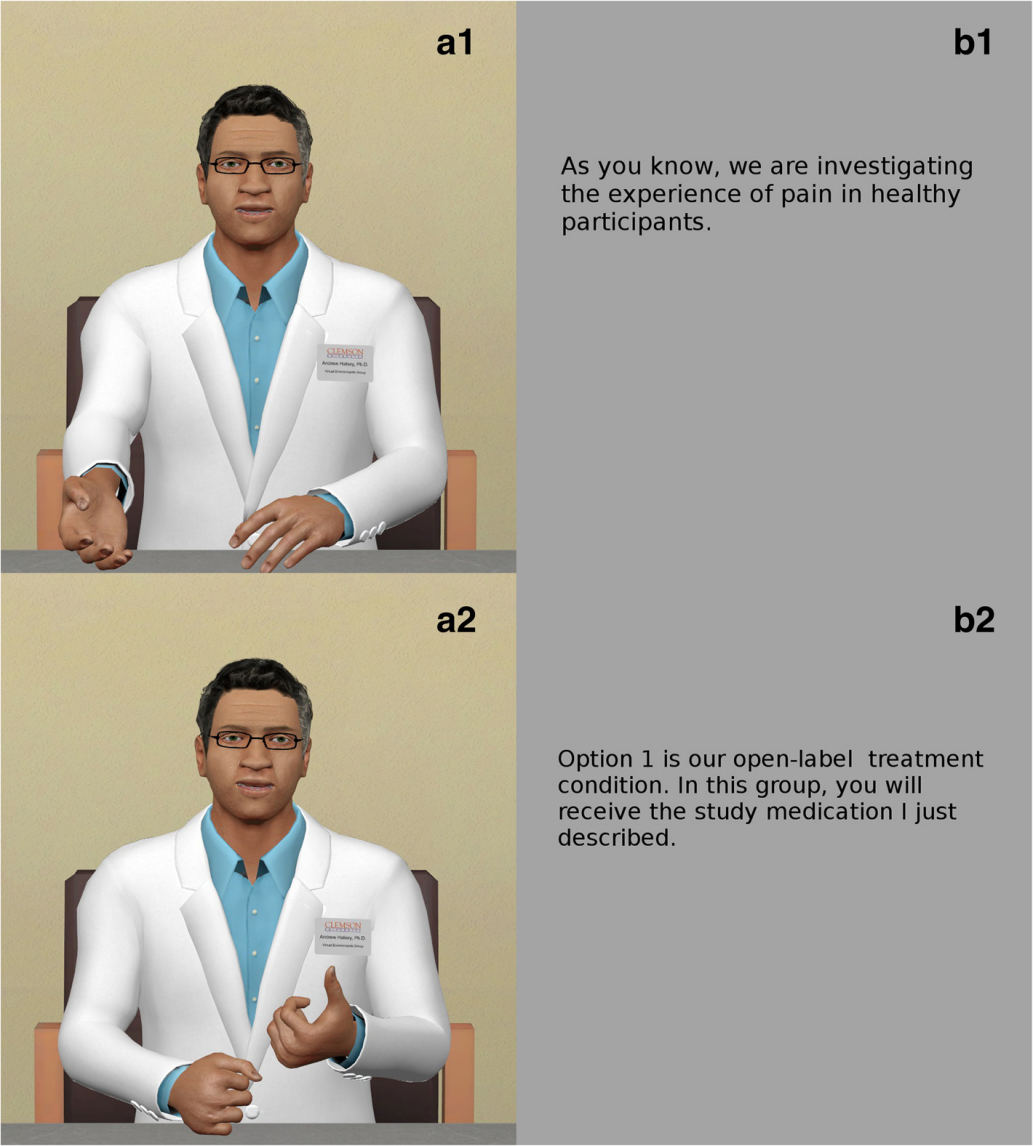
Visual display of two of the three Metaphors; the audio output is identical between both. **a1/a2** The VEx “Dr. Halsey” while gesturing. **b1/b2** Audio/text condition during the segments corresponding to **a1/a2**

The VEx behavior includes looped idle animations, conversation-triggered gestures, and facial expressions. Experimental instructions were prerecorded by a voice actor and lip-synchronized with the virtual agent. The audio/text Metaphor includes the same voice recording as the VEx Metaphor (Fig. 3a), but displays text instructions alongside the recording, instead of the virtual agent (Fig. 3b). This condition is included to gauge the relevance of visual social cues beyond the verbal content of the instruction.

Additional details pertaining to the software employed in the experiment is provided below (see section “Current status”).

### Predictor Instruction: placebo manipulation versus control

The placebo manipulation is contained in the Instruction received by the participant around the dispension of “medication”. It consists of the exchanging of a single sentence, stating either

*“you have been assigned to the open-label treatment condition. This means that the pills you will receive are definitely the study medication”* (in the “medication” Instruction)

or

*“you have been assigned to the open-label control condition. This means that the pills you will take are placebo pills, that is, contain only an inactive substance”* (in the “inert” Instruction).

To reinforce group allocation, two side effect questionnaires will be answered by the “medication” group only – one directly after placebo manipulation, the other at the end of the intermission. Furthermore, only “medication” group participants will be asked about their prior experience with the study drug, how effective it was in their experience, and how effective they assume it will be in reducing the pain at retest.

### Secondary measures

A number of putative predictors of pain sensitivity and placebo responsiveness [34] will be assessed before and during the experiment. These variables will be used as adjunct within- and between-persons predictors (covariates) of changes in pain sensitivity.

Psychological measures include pain questionnaires as well as several other psychological trait and state questionnaires (Table 2). Notably, participants will be asked about their desire for pain relief, and the expectation of pain relief after receiving the “medication” [5].

**Table 2:** Putative predictors of pain sensitivity and placebo responsiveness

Questionnaire/scale	Construct	Example item
Trait questionnaires, answered in online survey before coming to the lab		
Pain Related Self Statements inventory (PRSS)	Coping with pain, catastrophizing about pain	“If I stay calm and relax, things will be better.”
Pain Vigilance and Awareness Questionnaire (PVAQ)	Attentional focus on pain	“I am quick to notice changes in location or extent of pain.”
Pain Sensitivity Questionnaire (PSQ)	Sensitivity to various painful stimuli	“Imagine you trap your finger in a drawer.”
Beliefs about Medicines Questionnaire (BMQ)	Attitudes towards medical profession and pharmaceutics	“Medicines do more harm than good.”
Beliefs in Expectation Biases	Beliefs to what extent perception is influenced by expectations	“In general, people are likely to experience the mood (good or bad) they expect to experience.”
International Personality Item Pool Big Five scales (IPIP B5)	Extraversion, agreeableness, conscientiousness, emotional stability, intelligence/imagination	“I feel comfortable around people.”
Internality, Powerful Others, and Chance scales (IPC)	Attribution of events to personal control, other individuals, chance	“Whether people act according to my wishes depends mainly on myself.”
Revised Life Orientation Test (LOT-R)	Positive/negative expectation of outcomes = dispositional optimism/pessimism	“I’m always optimistic about my future.”
State questionnaires, answered during the lab visit		
State Trait Anxiety Inventory (STAI)	Acute and general anxiety	“I get in a state of tension or turmoil as I think over my recent concerns and interests.”
Multidimensional Mood Questionnaire (MDMQ)	Good/bad mood, calmness/nervousness, wakefulness/tiredness	“Right now I feel […] content.”

Physiological measures include heart rate as a proxy of general autonomic arousal [35] and high-frequency heart rate variability as a dedicated measure of parasympathetic withdrawal (cf. [36]). Furthermore, blood pressure [37] and skin temperature of the immersed hand [38] will be included as putative predictors of heat pain sensitivity. Heart rate and heart rate variability will be assessed with a Biolog device with Fetrodes technology (UFI, Morrow Bay, CA) during a 5 min physiological baseline, and during the measurements themselves (each sequence lasting 7.5 min). Blood pressure will be assessed with a calibrated GE Dinamap Pro100 (Medical Solutions, Minneapolis, MN) device. The last four of five measurements, spaced 1 min apart [39], will be averaged to derive systolic and diastolic blood pressure. Blood pressure will only be assessed during the physiological baseline, as cuff inflations could distract from the pain stimulation. Skin temperature will be assessed with an infrared thermometer before and after each Sequence, as well as at multiple time points during the intermission.

### Bias reduction and manipulation check

A manipulation check will be performed assessing any doubts or suspicions concerning the treatment allocation or the cover story. Preliminary data indicates that the cover story, including the group allocation, are considered convincing by the participants: 18 of 19 people answered “no” to a post-experimental question asking “Did you at any point find the experimental instructions unconvincing?”.

Allocation to the experimental groups will happen in a room adjacent to the room where the pain measurement takes place, and without insight of the laboratory assistant into the allocation. He or she will therefore be fully blinded to the treatment allocation when performing the retest. Measures will be taken to ensure that allocation is not revealed by the participant: The participant will be asked by the VEx, HEx or audio/text display not to notify the laboratory assistant of their treatment allocation, and the laboratory assistant will document any deviations. Audiovisual recording of the participant/laboratory assistant-interaction will be considered if necessary.

All measurements (except blood pressure and skin temperature) and pain reports will be performed on a computer without communication with the laboratory assistant. Furthermore, the instructions for the continuous pain ratings contain a line to emphasize participant anonymity and non-involvement of the laboratory assistant in data analysis, “This next part is very important: Your ratings will be analyzed by a blinded experimenter only after the session is over. Focus as much as possible on the sensation in your immersed hand, and feel totally free to rate your pain precisely and truthfully. Try not to let my presence or any other thought affect the way you report the sensation, or affect the number of trials you complete.” Also, during the pain measurement, the laboratory assistant will remove himself or herself from the immediate vicinity of the participant to sit down at a table in the participant's field of view, but facing in the same direction as the participant as to not give the participant the feeling of being observed (beyond an initial making sure that all instructions are complied with).

Some extent of selection bias is expected, as an aversive stimulation is employed. While a monetary compensation of $20 is offered for the ca. 2 h experiment, pain sensitive people will be potentially underrepresented in the sample. Systematic deviations from the norm will be assessed by analyzing questionnaire data (specifically, reported pain sensitivity via the PSQ) and actual responding to the stimulus. Preliminary data does not suggest that this is a serious issue, with roughly normally distributed pain sensitivity (data not shown).

### Analysis and sample size estimation

Data entry is almost fully automated via the touch-screen interface (for continuous pain ratings), a digital survey tool (for other pain ratings and psychological measurements), and on-line registration of physiological parameters. The exception are blood pressure and temperature data which are documented on a score sheet. Continuous pain ratings will be recorded at a sampling rate of ca. 40 Hz, but downsampled to 1 s means for analysis.

Data will be analyzed with hierarchical linear modelling (HLM). Among other strengths, this approach enables us to consider both categorical and continuous predictor variables (e.g., covariates), and the inclusion of cases with missing values, allowing for the number of repeated measures to differ across persons [40]. HLM puts no constraints on the number of repeated measurements, which permits an analysis of differences in the time course of pain. HLM also allows for considering random effects (intercepts and slopes), suitable to the high interindividual variability of pain sensitivity [41].

The robustness of results will be assessed by outlier analyses of the regressional models provided by HLM, including contrasting of predicted versus actual values, studentized residuals and Mahalanobis’ distance. Outliers will be excluded both at the within-person level (individual measurement occasions) and the between-person level (exclusion of individuals with outliers). Robustness will be gauged by changes in the significance of the results.

HLM sample size estimation considers the sample size at level 1 (*n*; in our case, the repeated measurements within-persons) and the sample size at level 2 (*N*; in our case, the individual participants). Recommendations vary (e.g. [42, 43]); Hox [43] suggests larger sample sizes of *n* = 20 and *N* = 50 when investigating cross-level interactions. The wealth of data points provided by the second-by-second continuous pain ratings easily allow for meeting the *n* = 20 suggestion. However, while a total of 600 s of immersions will be available, the effective sample size will be lower due to large intraclass correlations, or strong autocorrelation. Due to the high interindividual variation of pain sensitivity, we would nevertheless aim for including *N* = 50 individual participants to determine difference between the six groups. Estimating 15 % attrition, the projected sample size therefore amounts to *N* = 58 individuals.

### Hypotheses and expected results

The PE would be constituted by a larger symptom improvement from Sequence 1 to Sequence 2 in the “medication” Instruction, than in the “control/placebo” Instruction (“medication”(S2-S1)>“control”(S2-S1). This is a general test of the efficacy of the placebo paradigm, which is a crucial building block for assessing the effect of the different Metaphors.

The design does not strictly permit the establishment of equivalence between Metaphors in the sense of a non-inferiority trial, which is deemed too ambitious at this stage. For the proposed protocol, a non-significant difference between VEx and HEx would be considered as a first step to establish the viability of the VEx (HEx = VEx). On a descriptive level, we are expecting a slightly lower effect induced by the VEx, but lower variance than under the HEx, amounting to comparable effect sizes. Beyond that, it is hypothesized that the audio/text Metaphor induces a smaller symptom improvement than the other conditions (HEx = VEx > audio/text) due to absence of visual social cues.

**Fig. 4:**
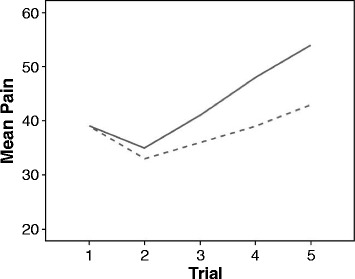
Possible results on the Trial level during Sequence 2 (post-intervention): Lower pain increase within the “medication” group (*dashed line*) vs. the “control” group (*solid line*)

Using a more fine grained analysis at the Trial level could yield additional information, such that groups differ in early or late Trials only, or that the slope (pain changes in consecutive Trials) is different between groups. For example, a significant difference between “medication” vs. “control” could come about by a shallower pain increase in consecutive Trials in Sequence 2, despite identical initial sensitivity (see Fig. 4).

If we indeed do not find significant differences of PEs induced by the VEx versus an HEx, we will proceed to more complex protocols (see “Potential and outlook”). If we do find differences such that an HEx proves superior to the VEx, we will return to the design phase and address weaknesses in the VEx software in an iterative design-test protocol until we can reliably elicit PEs using the VEx that are of comparable size with those elicited by an HEx.

#### Current status

In the following, the development of the virtual experimenter is described in more detail. Furthermore, preliminary results from a pilot study pertaining to the VEx software are summarized (Pilot study 1). Finally, results from a pilot study pertaining to the pain protocol are summarized (Pilot study 2).

### Development of the stimulus material

The design of the VEx “Dr. Halsey” (cf. Fig. 1) was informed by a preliminary survey including eight different photographs of supposed study physicians. Photographs were rated by a non-random sample (*N* = 10) on various dimensions (e.g., appearing competent and empathetic [44], trustworthy, believable, and enthusiastic [1, 18]) (data not shown).

The VEx prototype was modeled using MakeHuman version 1.0.1 and Blender version 2.69 (Blender Foundation, Amsterdam, Netherlands). It is rendered in a Unity 5.1.1 framework (Unity Technologies, San Francisco, CA, USA) on a 60“ screen display with 3D-capabilities. Lip-synchronization was performed using faceshift (faceshift, San Francisco, CA, USA). The software also includes an interaction module, which enables participants to ask the VEx a number of questions using a second 17” touch-screen display. Voice recognition is performed via the Microsoft Kinect (Microsoft, Redmond, WA) and processed using the Kinect Software Development Kit 1.8 (Microsoft, Redmond, WA) within the Unity framework rendering the VEx.

### Pilot study 1: evaluation of the virtual experimenter

This study was aimed at informing the direction of further development of the VEx, by investigating how the VEx prototype is perceived by random participants. The study protocol outlined above will employ a version of the piloted prototype once all changes according to the findings of this study have been integrated.

In this pilot study, 48 participants (23 female, 25 male; age mean ± SD 20.2 ± 2.3) watched the VEx instruction without actually undergoing the pain protocol, or receiving the presumed medication. Participants judged the VEx on an Expectancy Induction Characteristics questionnaire assessing dimensions considered conducive to placebo responses (e.g. [1, 18, 19]). The Expectancy Induction Characteristics questionnaire was constructed in our lab and consists of 11 items (semantic differentials). The items are subsumed in three scales pertaining to how convincing a person is perceived (scale Message), how likeable (scale Likeable), and how compelling (scale Compelling). The scales exhibit decent reliability with Cronbach’s alpha of .82, .68 and .82, respectively.

**Fig. 5:**
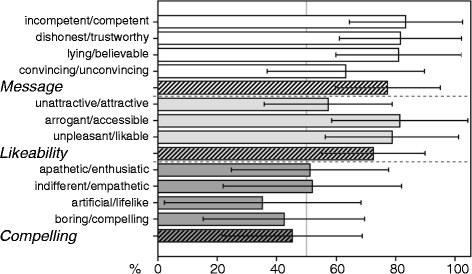
Rating (in percent) of the virtual experimenter simulation along 11 semantic differentials. Bars with thick borders display the scale means (*white* = Message, *light grey* = Likeability, *dark grey* = Compelling) composed of the items directly above

Results are presented in Fig. 5. While the VEx was judged favorably on the Message and Likeability scales, it received comparatively bad rating on the Compelling scale. We believe this to be owed to a rather neutral demeanor (both in verbal and nonverbal content) displayed by the model, which has been intentional to establish a solid baseline of the VEx’s efficacy. However, we are currently in the process of improving ratings in this scale by implementing higher behavioral realism (e.g. breathing, pre-recorded eye blinks) and a more animated presentation (e.g. more inflections during speech, more pronounced facial expressions).

### Pilot study 2: evaluation of the pain protocol

To characterize the pain protocol (see above) and estimate reproducibility across Sequences (including possible carry-over effects like habituation and sensitization), we conducted a pilot study with only a waiting period between the two Sequences. All means are provided with standard deviations; significance testing was performed with hierarchical linear model analysis, cf. “Analysis and sample size estimation” (a more in-depth discussion is beyond the scope of this article). In this study, we included 33 healthy participants (21 female, 12 male; age 20.1 ± 2.4). Continuous pain ratings almost invariably increased within Sequences/between Trials. On average, participants showed substantial amounts of pain in both Sequence 1 (VAS 46.4 ± 15.0, range 23.5 to 83.6) and Sequence 2 (VAS 40.8 ± 15.5, range 18.8 to 78.8), with normal distributions on both occasions. These means constitute a slight but significant overall decrease in pain between the two Sequences. Conversely, single retrospective ratings show no difference for intensity (VAS 53.8 ± 17.7 versus 52.6 ± 21.1) and unpleasantness (VAS 55.9 ± 17.1 versus 55.6 ± 21.3).

The continuous ratings exhibited good reproducibility (intraclass correlation coefficient(2,1) *ρ* = .72) between the two Sequences, comparable to that of the retrospective ratings of intensity (*ρ* = .77) and unpleasantness (*ρ* = .68).

## Discussion

### Challenges

The two core challenges are the establishment of PEs in a tightly controlled protocol with only limited “dosing” of the placebo manipulation, and achieving a sufficient comparability of the VEx with the HEx interaction as to mimic the psychosocial effects necessary for PEs to arise. The placebo manipulation only contains a verbal expectancy manipulation comparable to those used in other placebo experiments (e.g. [5]). However, we chose to limit emphasis and enthusiasm on the side of the experimenter as much as possible, as to introduce as few as possible confounding factors at this point. This may mean that the PE will not be pronounced.

With the VEx, the greatest challenge is to create a sufficient rapport between him and the participant to be perceived as a social situation. The Ethopoeia concept [45] posits a substrate neutrality of human versus virtual agent behavior: as long as social cues are displayed, they will lead to “mindless responses” [45], p. 83). However, these cues go beyond the behavioral realism of the display (e.g. facial expressions, gestures, blinking, breathing) – which is necessary but not sufficient [46] – and extent to interactional components to attribute agency to the virtual agent, such as turn-taking, visual attention to interlocutors, and behaviors reducing inter-personal distance. While technology like the “rapport agent” exists [47, 48], which has been tailored to naturally engage humans in conversation, our VEx software does not yet exhibit the same capabilities. At this stage, the VEx includes an FAQ module, engaging participants to ask questions with prerecorded answers; furthermore, verbal input will be required from the participants at certain points of the protocol to continue (cf. Fig. 3b). However, in future iterations, actually artificial intelligence-driven conversation is planned [49], engaging participants in personal questions e.g. about demographic variables in a small talk/rapport module.

As to the appearance of the VEx, a balance will be struck between realism and acceptance – specifically, the design has to avoid the so-called “uncanny valley” [50] describing an aversive perception of almost-but-not-quite realistic artificial agents by human spectators.

### Limitations

The rationale of using the VEx in the context of experimental studies makes the assumptions that the treatment delivery and the accompanying instructions are, in fact, the most important aspects for the generation of PRs. While instructions alone have been shown to effect PEs [5, 51, 52], it has been demonstrated that conditioning paradigms may have better results [6]. However, a conditioning approach was considered too logistically demanding at this stage.

It is possible that the control group itself will not fully believe in the veracity of them only receiving inert medication. However, this would lead to a conservative estimate of placebo effectiveness and is therefore no hazard to the internal validity of the study. We are also considering the option of using a no-treatment control (i.e., no placebo delivery at all) instead of a “control/placebo” instruction.

While evidence exists that female doctors are estimated to have better explanation skills and technical skills and have a higher acceptance [53], the decision to only include male models in the creation of the VEx was made for opportunistic reasons (having a male voice actor available).

On a more general note, while the main intention behind the creation of the VEx is the assessment and abolishment of biases in psychological and medical research, there is a possibility that the use of a virtual agent would induce biases of another sort, such as involving computer (il)literacy or opinions towards technological progress [54]. For example, it is feasible that older participants would have a different response to the VEx’ than younger participants, who will have been exposed to virtual reality technology to a higher extent. On the plus side, there is evidence that participants tend to disclose more personal information to a virtual agent than a human experimenter [55, 56], which may be of relevance with increased interactional capabilities of the VEx. Nevertheless, generalizability will be attempted by using a sample beyond student participants. We are further addressing these issues by post-experiment questionnaires and demographical data about the experience with, and daily use of computers and 3D technology.

Furthermore, the novelty of the simulation may serve to accentuate or alleviate effects which would otherwise be (in)effective. The experience of novelty will be assessed and investigated as a potential moderator of treatment efficacy.

### Potential and outlook

The broader objective of investigating the VEx is to estimate its comparability to a real human. Using a placebo paradigm is considered an optimal testbed of this comparability due to its dependence on psychosocial variables. Regardless of outcome pertaining to the efficacy of the VEx, results from this study will be disseminated to a wider public, since they will contain valuable information on the specific mechanisms involved in the generation of PEs. Success of the study therefore does not hinge on the efficacy of the VEx, only on replicating results from previous placebo manipulations by the HEx.

Should it be established that the VEx can, in fact, serve as a substitute to an HEx, the possible applications in medical and general psychological research are many. Furthermore, it should be kept in mind that while a VEx would prove to be of great value for placebo research, applicability goes far beyond this narrow topic and extends into most forms of psychological experiments where standardization is of relevance. These applications fall into three interrelated categories – standardization, experimental modification, and communication. To these ends, we are making efforts to develop the software such that it can be easily adopted by other researchers, for whom it would eventually be made available.

Standardization of experimenter characteristics would facilitate the investigation of psychological entities feasibly affected by psychosocial interactions. As no repeated acting/performance would be required, a VEx could serve as a “perfect confederate” [57], p. 6) in social psychological studies, or studies involving social learning. A VEx would get rid of experimenter-side biases due to unblinding, especially if being a stakeholder in an experiment’s success or other factors would incentivize an HEx to guess treatment allocations or behave differently according to which group one is assigned to, introducing experimenter demand characteristics [58].

For placebo research, the putative role of experimenter variables has been elaborated above. Yet, these factors have been underreported [59], introducing confounders and thus exacerbating the problems created under non-blind conditions. One reason for this is the difficulty of assessing or standardizing these factors. Not only could a VEx serve as a perfectly standardized platform if these factors are sought to be held constant: Instead, it would provide a convenient means to modify single aspects of the experimenter characteristics while holding all others equal – such as gender, age or professional role of the experimenter, or informational content such as the phrasing of instructions. This would not only inform the respective research (e.g. about placebo mechanisms), but also the creation of virtual agents used as interfaces in customer relations and medical devices.

Furthermore, with a VEx interface, participant behavior could be monitored much easier, for example by logging which questions are asked by the participant. While it will strongly depend on the concrete experimental context if a VEx can exhaustively serve the informational needs of a participant, pilot studies could inform the formulation of frequently asked questions to be covered by the VEx.

Lastly, the standardization aspect not only applies to single experiments, but to collaborative, multi-center research efforts as well. Since the communication effectively only entails distribution of software, it could serve as an easy means to establish instruction equivalence. This is particularly relevant considering that insufficient reliability poses serious economic and scientific problems for randomized controlled trials [60, 61], with even double-blind conditions being no safeguard against yielding biased results [62]. It is conceivable that outsourcing suitable study tasks (like consent, instructions, medication, questionnaire administration) would free up resources which could be spent on other activities, reducing a trial’s logistical burden. The VEx could be used as best practice example or in training videos. It could contribute to advances in telemedicine or similar situations where a virtual human interface has been shown to increase usability, e.g., with elderly people [63]. With the preservation of social cues, the possibility of instruction delivery via different telecommunication devices could prove invaluable for ambulatory treatment where improvement in compliance and treatment efficacy are sought.

## Abbreviations

FAQ, frequently asked questions; HEx, human experimenter; HLM, hierarchical linear modelling; PE, placebo effect; VAS, visual analogue scale; VEx, virtual experimenter
